# A Validated HPLC-MS/MS Method for Simultaneous Determination of Militarine and Its Three Metabolites in Rat Plasma: Application to a Pharmacokinetic Study

**DOI:** 10.1155/2019/2371784

**Published:** 2019-05-02

**Authors:** Hui-Yuan Sun, Lin Zheng, Zi-Peng Gong, Yue-Ting Li, Chang Yang, Jie Pan, Yong-Lin Wang, Ai-Min Wang, Yong-Jun Li, Yong Huang

**Affiliations:** ^1^Guizhou Provincial Key Laboratory of Pharmaceutics, State Key Laboratory of Functions and Applications of Medicinal Plants, Guizhou Medical University, Guiyang 550004, China; ^2^School of Pharmacy, Guizhou Medical University, Guiyang 550004, China; ^3^Guizhou Provincial Engineering Research Center for the Development and Application of Ethnic Medicine and TCM, Guizhou Medical University, Guiyang 550004, China

## Abstract

A rapid, reliable, and sensitive HPLC-electrospray ionization-tandem mass spectrometry (HPLC-MS/MS) method was established and validated for simultaneous determination of militarine and its three metabolites (gastrodin, *α*-isobutylmalic acid, and gymnoside I) in rat plasma. Plasma was acidified with formic acid, and protein was precipitated with methanol. MS/MS with ESI and multiple reaction monitoring at* m/z* 725.3→457.3, 457.1→127, 304.3→107.2, 189→129, and 417.1→267.1 was used for determination of militarine, gastrodin, *α*-isobutylmalic acid, gymnoside I, and puerarin (internal standard), respectively. Chromatographic separation was conducted using an ACE UltraCore SuperC18 (2.1 × 100 mm, 2.5 *μ*m) column with gradient mobile phase (0.1% formic acid in water and acetonitrile). The lower limits of quantitation for militarine, gastrodin, *α*-isobutylmalic acid, and gymnoside I were 1.02, 2.96, 1.64, and 0.3 ng/mL, respectively. The relative standard deviations of intra- and interday measurements were less than 15%, and the method accuracy ranged from 87.4% to 112.5%. The extraction recovery was 83.52%-105.34%, and no matrix effect was observed. The three metabolites (gastrodin, *α*-isobutylmalic acid, and gymnoside I) were synchronously detected at 0.83 h, suggesting that militarine was rapidly transformed to gastrodin, *α*-isobutylmalic acid, and gymnoside I. Moreover, the area under the curve (AUC) and C_max_ of militarine were significantly lower than those of gastrodin and *α*-isobutylmalic acid, showing that militarine was largely metabolized to gastrodin and *α*-isobutylmalic acid* in vivo*. The studies on pharmacokinetics of militarine and its three metabolites were of great use for facilitating the clinical application of militarine and were also highly meaningful for the potential development of militarine.

## 1. Introduction

**Figure 1:**
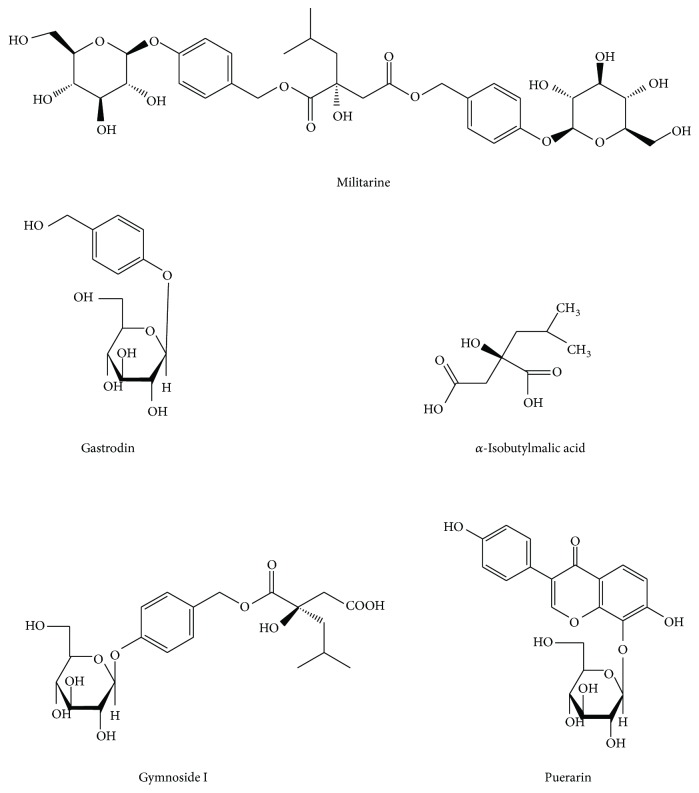
The chemical structures of gastrodin, *α*-isobutylmalic acid, gymnoside I, militarine, and puerarin (IS).

Militarine (Figure 1), a natural glucosyloxybenzyl 2-isobutylmalate containing 2-isobutylmalate as a parent nucleus, is widely distributed in orchids (Orchidaceae) [1], such as* Coeloglossum viride (L.)* Hartm. var.* bracteatum* [2] and* Bletilla striata* (Thunb.) Reichb. f. [3]. Recent pharmacological studies have shown that glucosyloxybenzyl 2-isobutylmalates had antiaging and neuroprotective effects; additionally, they could improve intelligence, learning ability, and memory and prevent senile dementia [4–8]. Militarine is the most abundant active component in* B. striata* (Thunb.) Reichb. f. [9–12].* B. striata* (Thunb.) Reichb. f. has been widely used for the treatment of traumatic bleeding, hemoptysis, and hematemesis owing to its astringent and antihemorrhagic effects. In addition, it has been applied topically to relieve sores, ulcers, chapped skin, and swelling owing to its tissue regenerative capabilities [13–17].* B. striata* is a commonly used traditional Chinese medicine (TCM) [18, 19]. Moreover, the vasodilator and learning and memory-improving effects of militarine have been verified [20, 21]. However, only few studies have investigated the potential effects of militarine at present.

To better understand and elucidate the relationship between the pharmacological actions and biotransformation of militarine* in vivo*, a good knowledge of the ADME processes of this constituent is getting more essential. A rat study on the pharmacokinetics of militarine (21.51 mg/kg, i.g.) suggested that the plasma concentration (C_max_ = 30.3 ± 8.61 ng/mL, AUC_0-t_ = 74.3 ± 37.68 h*∗*ng/mL) of militarine was low [18]. The militarine was therefore speculated to be intensely metabolized in vivo in this study. However, the metabolism of militarine has not been reported in the literature as far as we know. It has been shown that militarine can be metabolized into gastrodin, *α*-isobutylmalic acid, and gymnoside I in our previous studies. Therefore, to understand the pharmacokinetics of militarine and its metabolites* in vivo*, a quantitative method was established to determine the dynamic changes of militarine and its metabolites* in vivo*.

To simultaneously determine militarine and its metabolites in rat plasma, a sensitive bioanalytical method should be employed. In addition, the analytical method should be selective to avoid interference from endogenous substances in the plasma. High-performance liquid chromatography-tandem mass spectrometry (HPLC-MS/MS) with multiple reaction monitoring (MRM) mode utilizes two stages of mass filtering, which can specifically measure the relative and absolute analyte concentrations [22]. Thus, HPLC-MS/MS could be used for quantitative analysis of drugs and their metabolites.

In this study, a rapid, reliable, and sensitive HPLC-MS/MS method was developed and validated for the simultaneous determination of militarine and its three metabolites (gastrodin, *α*-isobutylmalic acid, and gymnoside I) in rat plasma to study the pharmacokinetics of militarine after single oral administration to rats. To the best of our knowledge, this is the first study to determine the pharmacokinetics of militarine and its three metabolites, which could be highly meaningful for the potential development of militarine.

## 2. Materials and Methods

### 2.1. Reagents and Chemicals

Militarine (purity ≥ 98%) was purchased from Chengdu Push Bio-Technology Co., Ltd. (Chengdu, China). Gastrodin (purity: 97.6%) and puerarin (IS, purity: 95.4%) were obtained from the National Institutes for Food and Drug Control (Beijing, China). Gymnoside I (purity ≥ 95%) and *α*-isobutylmalic acid (purity ≥ 95%) were isolated from* B. striata* (Thunb.) Reichb. f. in our laboratory. The structure and purity of these compounds were confirmed using IR, ^1^H nuclear magnetic resonance (NMR), MS, and HPLC-UV. Their chemical structures are shown in Figure 1. Methanol, formic acid, and acetonitrile of HPLC-grade were purchased from Merck KGaA Co. (Darmstadt, Germany). Deionized water was obtained using an EPED superpurification system (EPED, Nanjing, China). Other reagents and chemicals were of chromatographic grade.

### 2.2. Animals

Sprague-Dawley rats (250 ± 20 g) were supplied by Changsha Tianqin Biotechnology Co., Ltd. (Changsha, China, certificate No. SCXK (Xiang) 2014-0010). All studies were approved by the Animal Ethics Committee at Guizhou Medical University.

### 2.3. Instrumentation and Analytical Conditions of HPLC-MS/MS

An Acquity HPLC system (Shimadzu Corp., Kyoto, Japan) equipped with a Q-Trap ® 5500 triple quadruple mass spectrometer (AB Sciex, Foster, CA, USA) was used for HPLC-ESI-MS/MS. Applied Biosystems Analyst software version 1.6.2 was used for data acquisition. The four analytes and the IS (puerarin) were chromatographically separated using an ACE UltraCore SuperC18 (2.1 × 100 mm, 2.5 *μ*m) column, and the column temperature was set at 45°C. The mobile phase was a binary solvent system, consisting of 0.1% (v/v) formic acid aqueous solution (A) and acetonitrile (B) at a flow rate of 0.2 mL/min. Gradient elution was used as follows: 10-10% B at 0-1 min, 10-30% B at 1-2 min, 30-40% B at 2-6 min, 40-90% B at 6-6.1 min, 90-90% B at 6.1-8.1 min, 90-10% B at 8.1-8.2 min, and 10-10% B at 8.2-12 min. The injection volume was 1 *μ*L.

Detection of the analytes was carried out simultaneously with electrospray negative ionization (ESI^–^) and electrospray positive ionization (ESI^+^), and high-purity nitrogen served as both the nebulizing and drying gas. The optimized parameters were as follows: curtain gas (CUR), 0.28 MPa; ion source gas 1 (GS1), 0.38 MPa; ion source gas 2 (GS2), 0.38 MPa; source temperature, 500°C; and spray voltages, 5500 V (ESI^+^) and 4500 V (ESI^−^). The optimized MS parameters are listed in Table 1.

### 2.4. Preparation of Calibration Standards and QC Sample

The stock solutions of militarine, gastrodin, *α*-isobutylmalic acid, and gymnoside I were separately weighed and dissolved in methanol to obtain final concentrations of 1.8, 1, 2.9, and 1.1 mg/mL, respectively. Four series of standard mixture working standard solutions were obtained by mixing and diluting the respective stock solutions with methanol. An appropriate amount of puerarin was dissolved in methanol and diluted to obtain the IS solution (20 ng/mL). The mixture working standard solution (50 *μ*L) and IS solution (20 *μ*L) were added to blank rat plasma (100 *μ*L) to prepare the calibration standard solutions at final concentrations of 1.02-97.92, 2.96-1136.64, 1.64-629.76, 0.3-57.6, and 4 ng/mL for militarine, gastrodin, *α*-isobutylmalic acid, gymnoside I, and the IS, respectively. The QC plasma samples, containing militarine (2.04, 32.64, and 97.92 ng/mL), gastrodin (5.92, 378.88, and 1136.64 ng/mL), *α*-isobutylmalic acid (3.28, 209.92, and 629.76 ng/mL), and gymnoside I (0.6, 19.2, and 57.6 ng/mL), were prepared in the same manner.

### 2.5. Sample Preparation

An aliquot (100 *μ*L) of rat plasma, 20 *μ*L of IS solution (20 ng/mL), and 40 *μ*L of 1% formic acid solution were added into a 1.5-mL centrifuge tube and vortexed for 30 s. The mixture was extracted with 400 *μ*L of methanol by shaking for 2 min using a vortex mixer. After centrifugation at 13225 ×*g* for 10 min at 4°C, the supernatant was quantitatively transferred to a clean centrifuge tube and evaporated to dryness under a stream of nitrogen at 37°C. The residue was redissolved with 200 *μ*L of 50% methanol and centrifuged at 13225 ×*g* for 10 min. Then, the supernatant (1 *μ*L) was used for analysis.

### 2.6. Method Validation

Before using the proposed method to determine militarine, gastrodin, *α*-isobutylmalic acid, and gymnoside I in plasma samples, the method was fully validated for specificity, selectivity, linearity, LLOQ, precision, accuracy, extraction recovery, matrix effect, and stability according to the nonclinical drug pharmacokinetic study technical guideline (China Food And Drug Administration 2014) and the Bioanalytical Method Validation Guideline (Chinese Pharmacopoeia 2015, Vol. 4).

#### 2.6.1. Specificity and Selectivity

Specificity and selectivity were assessed by comparing the chromatograms of blank plasma from six different rats, blank plasma spiked with militarine, gastrodin, *α*-isobutylmalic acid, gymnoside I and the IS, and plasma samples obtained after oral administration of militarine at 60 mg/kg.

#### 2.6.2. Linearity and LLOQ

Calibration curves were constructed, as described in Section 2.4. Linearity was evaluated by plotting the peak area ratio (*y*) of analytes to IS versus the nominal concentration (*x*) of analytes by using 1/x^2^ weighted least squares linear regression. The LLOQ should satisfy the analytical requirement of a signal-to-noise ratio (S/N) of approximately 10.

#### 2.6.3. Precision and Accuracy

Precision and accuracy were determined by analyzing the QC samples in five replicates at three concentration levels (namely, low, medium, and high) on the same day (intraday) and on three consecutive validation days (interday).

#### 2.6.4. Extraction Recovery and Matrix Effect

The extraction recovery of the analytes was evaluated by comparing the peak area ratios of pretreated QC samples at low, medium, and high concentrations with those of post-extracted supernatants spiked with the pure reference standards at the same concentrations. The matrix effect was assessed by comparing the peak areas of the analytes in the post-extracted spiked samples with those of the analytes dissolved in methanol at the same concentrations. Analysis of the QC samples was performed in five replicates.

#### 2.6.5. Stability

The stability of analytes was determined using the QC samples at low, medium, and high concentrations (*n *= 5) under various conditions: post-preparation stability (24°C in an autosampler for 6 h) and freeze-thaw stability (three times, -20°C to 20°C) on three consecutive days.

### 2.7. Pharmacokinetic Study

In the pharmacokinetic experiment, the validated method was used to determine the plasma concentrations of militarine and its three metabolites (gastrodin, *α*-isobutylmalic acid, and gymnoside I) in six healthy SD rats weighing 250 ± 20 g. Rats were fasted with free access to water for 12 h before the experiment. They received a single oral dose (60 mg/kg) of militarine. Blood samples of 300 *μ*L were collected from the external right jugular vein into heparinized tubes at the designated time points (0, 0.033, 0.083, 0.167, 0.33, 0.5, 1, 1.5, 2, 4, 6, 8, 12, 24, and 36 h). The heparinized blood samples were centrifuged at 3306 ×*g* for 10 min at 4°C, and the supernatant was transferred into clean centrifuge tubes and stored at -20°C until analysis.

### 2.8. Pharmacokinetic Data Analysis

The pharmacokinetic parameters of the four analytes were calculated by Phoenix WinNonlin version 6.4 software (Pharsight Corporation, Mountain View, USA) using noncompartmental analysis. These parameters included the AUC from 0 h to the time of last measurable concentration (AUC_0-t_), AUC from 0 to infinity (AUC_0–*∞*_), MRT_0–t_, MRT to infinity (MRT_0-*∞*_), C_max_, and time to C_max_ (T_max_). All results were expressed as the means ± standard deviations (SD).

## 3. Results and Discussion

### 3.1. Method Validation

#### 3.1.1. Specificity and Selectivity

**Figure 2:**
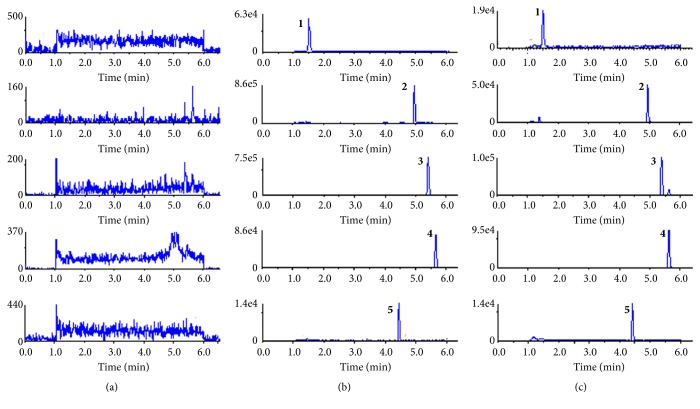
The chromatograms of the analytes and puerarin (IS) in rat plasma. (a) A blank rat plasma sample; (b) a blank rat plasma sample spiked with gastrodin (378.88 ng/mL), *α*-isobutylmalic acid (209.92 ng/mL), gymnoside I (19.2 ng/mL), militarine (32.64 ng/mL) and IS (4 ng/mL); (c) a plasma sample at 30 min after oral administration militarine to rats. (1) Gastrodin, (2) *α*-isobutylmalic acid, (3) gymnoside I, (4) militarine, and (5) puerarin (IS).

The chromatograms of blank rat plasma, blank rat plasma spiked with standard solutions and IS, and rat plasma after oral militarine administration are shown in Figure 2. The retention times of gastrodin, *α*-isobutylmalic acid, gymnoside I, militarine, and the IS (puerarin) were 1.52, 4.96, 5.4, 5.65, and 4.45 min, respectively. No significant interference from endogenous substances with the analytes and IS was observed, compared to the chromatogram of blank rat plasma sample.

#### 3.1.2. Linearity and Lower Limit of Quantitation (LLOQ)

Table 2 shows the typical calibration curves, linearity ranges, coefficients of correlation, and LLOQ for militarine, gymnoside I, gastrodin, and *α*-isobutylmalic acid. All the four analytes exhibited good linearity with correlation coefficients within the range of 0.9994-0.9997.

#### 3.1.3. Precision and Accuracy

The intra- and interday precision and accuracy were evaluated in five replicate analyses of quality control (QC) samples at three concentrations. Results are summarized in Table 3. The relative standard deviations of intra- and interday measurements were less than 15%, and the RSD (%) values of accuracy of three analytes were within the range of 87.4-112.5%. These results showed that the method was acceptable.

#### 3.1.4. Extraction Recovery and Matrix Effect

The mean extraction recovery and matrix effect of the four analytes are shown in Table 4. The extraction recoveries of the four analytes were within the range of 83.52-105.34%, showing that the recovery of analytes was consistent and reproducible. The matrix effects ranged from 84.42% to 102.82%. The results indicated that no endogenous substances significantly influenced the quantification of all the analytes.

#### 3.1.5. Stability

Table 5 shows the stability data of the four analytes. Results showed that the relative standard deviations (RSD) of post-preparation stability (24°C in an autosampler for 6 h) and freeze-thaw stability (three times, -20 to 20°C) on consecutive three days of all analytes were ≤ 9.6% and ≤ 9.9%, respectively. These results indicated that the four analytes were stable and applicable within the acceptable limit.

### 3.2. Pharmacokinetics

**Figure 3:**
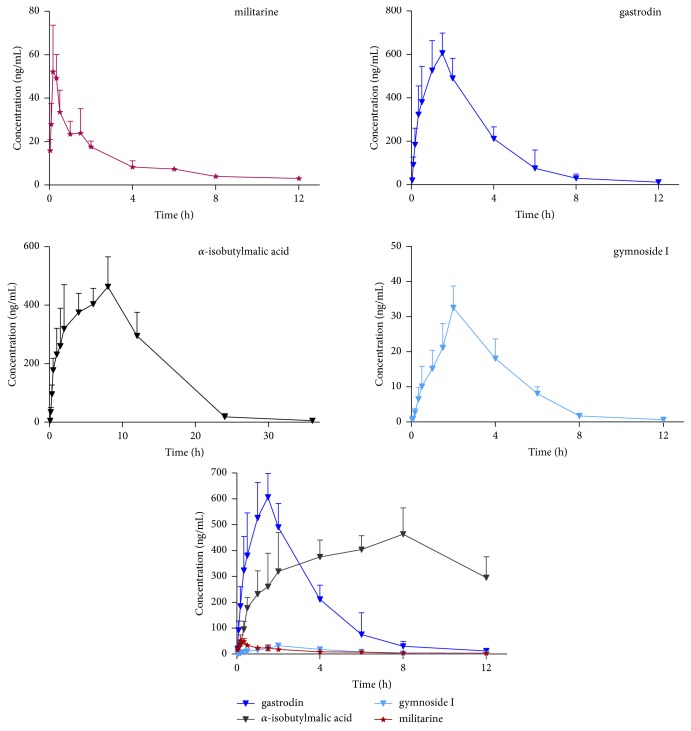
Mean plasma concentration-time curves of militarine and its three metabolites after the single oral dose (60 mg/kg) of militarine to rats (n=6).

The validated HPLC-MS/MS method was successfully applied for simultaneous determination of militarine and its three metabolites after single oral administration of militarine at 60 mg/kg to six healthy rats. The primary pharmacokinetic parameters calculated using noncompartmental analysis are summarized in Table 6. The mean plasma concentration-time curves of militarine and its three metabolites are shown in Figure 3.

Two minutes after administration, militarine could be detected in plasma, indicating that it was rapidly absorbed. Militarine reached the peak plasma concentration (C_max_ = 62.31 ± 10.01 ng/mL) at approximately 0.21 h and was rapidly eliminated with a mean retention time to the last sampling time (MRT_0-t_) of 3.29 ± 0.36 h. The three metabolites (gastrodin, *α*-isobutylmalic acid, and gymnoside I) were synchronously detected at 0.83 h, suggesting that militarine was rapidly transformed to gastrodin, *α*-isobutylmalic acid, and gymnoside I. Moreover, the area under the curve (AUC) and C_max_ of militarine were significantly lower than those of gastrodin and *α*-isobutylmalic acid, showing that militarine was largely metabolized to gastrodin and *α*-isobutylmalic acid* in vivo*. The AUC and C_max_ of gymnoside I were comparable to that of militarine. These results suggested that the cleavage of ester bonds in militarine could produce gymnoside I and gastrodin, and then the cleavage of the ester bonds in gymnoside I could produce gastrodin and *α*-isobutylmalic acid, which could account for the high AUC and C_max_ values of gastrodin and *α*-isobutylmalic acid, compared to those of militarine and gymnoside I. The order of AUC values was *α*-isobutylmalic > gastrodin > militarine > gymnoside I, suggesting that militarine could be adequately transformed to the three metabolites* in vivo*, and the most abundant constituents in the blood were *α*-isobutylmalic acid and gastrodin.

A drug candidate should have a favorable pharmacokinetic behavior to allow new drug development [23]. Militarine was shown to improve white matter lesions (WMLs) and cognitive impairment in rat chronic hypoperfusion model; additionally, it showed a dose-dependent and endothelium-dependent relaxing effect in rat isolated thoracic aorta rings [20, 21]. However, our results showed that the blood concentration of militarine was very low after oral administration, and gastrodin and *α*-isobutylmalic acid were the main components detected in the plasma after single oral administration (60 mg/kg) of militarine to rats. Gastrodin, the main bioactive ingredient in the widely known Chinese medicine “Tianma” (*Rhizoma Gastrodia*), has been widely used in China to treat central nervous system (CNS) diseases, such as neuralgia, vertigo, neurasthenia, epilepsy, insomnia, and headache [24–28]. Additionally, it has been shown to improve learning and memory and to exhibit vasodilator effects [29, 30]. *α*-Isobutylmalic acid exhibited longer MRT and higher C_max_ than those of militarine, indicating that *α*-isobutylmalic acid might exert therapeutic effects* in vivo*. In addition, glucosyloxybenzyl 2-isobutylmalates, such as gymnoside I, could improve intelligence and prevent senile dementia [1, 31]. Therefore, further studies are required to verify these effects of gymnoside I. The* in vivo* effectiveness of bioactive constituents depends to a great extent on their blood concentration, as well as the activity of their metabolites. Thus, the effects of militarine* in vivo* might be, in part, attributed to the activity of its metabolites. We believe that the results of this pharmacokinetic study of militarine after its oral administration in rats could provide the basis for the development of militarine and its clinical applications.

## 4. Conclusions

In this study, a sensitive, rapid, and convenient HPLC-MS/MS method to determine militarine and its three metabolites (gastrodin, *α*-isobutylmalic acid, and gymnoside I) in rat plasma was successfully established and validated. The validated method was successfully used in a preclinical pharmacokinetic study in rats after oral administration of militarine. This study determined the pharmacokinetic behavior of militarine and its metabolites. The current pharmacokinetic study of militarine not only provided significant and valuable clinical guidance toward the application of this constituent but also could predict the druggability of bioactive metabolites, which is essential in drug development.

## Figures and Tables

**Table 1:** Mass spectra properties of the analytes and IS.

Analytes	Precursor ion (m/z)	Product ion (m/z)	Declustering potential (DP, V)	Entrance potential (EP, V)	Collision energy (CE, V)	Cell exit potential (CXP, V)
gastrodin	304.3	107.2	57	8	23	18
*α*-isobutylmalic acid	189.0	129.0	-73	-10	-23	-13
gymnoside I	457.1	127.0	-77	-5	-32	-6
militarine	725.3	457.3	-43	-9	-22	-40
puerarin (IS)	417.1	267.1	110	7	35	6

**Table 2:** Calibration curves, linear ranges, correlation coefficients, and LLOQ of gastrodin, *α*-isobutylmalic acid, gymnoside I, and militarine in rat plasma (n=3).

Analytes	Linear regression equation	R^2^	Linear range (ng /mL)	LLOQ (ng /mL)
militarine	Y=0.0461X - 0.0924	0.9997	1.02 - 97.92	1.02
gastrodin	Y=0.4226X + 0.0112	0.9996	2.96 - 1136.64	2.96
*α*-isobutylmalic acid	Y=0.2270X + 0.0174	0.9994	1.64 - 629.76	1.64
gymnoside I	Y=0.0436X - 0.0093	0.9996	0.3 - 57.6	0.3

**Table 3:** Summary of precision and accuracy of the four analytes in rat plasma (n=5).

Analytes	Spiked Concentration (ng/mL)	Intra-day			Inter-day		
		Calculated concentration (ng/mL)	Precision (RSD, %)	Accuracy (%)	Calculated concentration (ng/mL)	Precision (RSD, %)	Accuracy (%)
militarine	2.04	2.22 ± 0.15	6.9	108.9	1.87 ± 0.21	11.4	91.5
	32.64	31.56 ± 2.37	7.5	96.7	36.73 ± 1.87	5.1	112.5
	97.92	105.10 ± 4.67	4.4	107.3	87.36 ± 5.97	6.8	89.2
gastrodin	5.92	6.48 ± 0.69	10.6	109.5	6.51 ± 0.63	9.7	109.9
	378.88	341.90 ± 18.12	5.3	90.2	335.42 ± 25.02	7.5	88.5
	1136.64	1253.71 ± 44.13	3.5	110.3	1051.16 ± 128.45	12.2	92.5
*α*-isobutylmalic acid	3.28	3.05 ± 0.38	12.5	93.0	3.59 ± 0.37	10.3	109.4
	209.92	230.72 ± 13.11	5.7	109.9	217.31 ± 27.23	12.5	103.5
	629.76	672.08 ± 49.73	7.4	106.7	575.98 ± 51.20	8.9	91.5
gymnoside I	0.6	0.58 ± 0.05	8.3	96.6	0.52 ± 0.05	10.1	87.4
	19.2	16.99 ± 1.97	11.6	88.5	17.53 ± 2.32	13.2	91.3
	57.6	52.70 ± 2.82	5.4	91.5	61.33 ± 5.70	9.3	106.5

**Table 4:** Summary of recovery and matrix effect of the four analytes in rat plasma (n=5).

Analytes	Spiked Concentration (ng/mL)	Extraction Recovery		Matrix effect	
		Mean ± SD (%)	RSD (%)	Mean ± SD (%)	RSD (%)
militarine	2.04	105.34 ± 7.91	7.5	102.82 ± 8.53	8.3
	32.64	92.40 ± 10.65	11.5	87.61 ± 11.49	13.1
	97.92	89.92 ± 8.99	10.0	92.52 ± 4.75	5.1
gastrodin	5.92	85.71 ± 6.01	7.0	88.80 ± 9.22	10.4
	378.88	92.49 ± 5.40	5.8	85.43 ± 4.63	5.4
	1136.64	87.58 ± 8.20	9.4	92.76 ± 6.78	7.3
*α*-isobutylmalic acid	3.28	93.14 ± 3.56	3.8	93.48 ± 8.07	8.6
	209.92	89.10 ± 10.88	12.2	84.42 ± 10.91	12.9
	629.76	95.71 ± 10.17	10.6	89.30 ± 10.13	11.3
gymnoside I	0.6	93.02 ± 3.53	3.8	85.41 ± 3.42	4.0
	19.2	90.40 ± 12.08	13.4	90.14 ± 8.92	9.9
	57.6	83.52 ± 2.86	3.4	88.30 ± 10.78	12.2

**Table 5:** Stability of the four analytes in rat plasma under various storage conditions (n = 5).

Analytes	Spiked Concentration (ng/mL)	Post-preparation stability			Freeze-thaw stability		
		Calculated concentration (ng/mL)	Precision (RSD, %)	Accuracy (%)	Calculated concentration (ng/mL)	Precision (RSD, %)	Accuracy (%)
militarine	2.04	1.85 ± 0.14	7.3	90.6	2.09 ± 0.21	9.9	102.6
	32.64	33.47 ± 2.22	6.6	102.5	35.09 ± 1.41	4.0	107.5
	97.92	96.57 ± 5.42	5.6	98.6	95.68 ± 7.57	7.9	97.7
gastrodin	5.92	6.29 ± 0.60	9.6	106.2	6.11 ± 0.59	9.6	103.2
	378.88	357.97 ± 11.85	3.3	94.5	410.63 ± 26.77	6.5	108.4
	1136.64	1080.94 ± 52.32	4.8	95.1	1087.88 ± 31.11	2.9	95.7
*α*-isobutylmalic acid	3.28	3.03 ± 0.15	4.9	92.4	3.21 ± 0.27	8.5	97.9
	209.92	213.51 ± 6.62	3.1	101.7	213.32 ± 13.89	6.5	101.6
	629.76	647.02 ± 27.63	4.3	102.7	607.66 ± 32.21	5.3	96.5
gymnoside I	0.60	0.62 ± 0.05	8.6	103.6	0.63 ± 0.05	8.5	104.6
	19.2	18.65 ± 1.34	7.2	97.1	20.14 ± 1.49	7.4	104.9
	57.6	54.29 ± 3.67	6.8	94.3	54.50 ± 1.91	3.5	94.6

**Table 6:** The pharmacokinetic parameters of militarine and its three metabolites after the single oral dose (60 mg/kg) of militarine to rats (n = 6, mean ± SD).

Parameters	Unit	Analytes			
		militarine	gastrodin	*α*-isobutylmalic acid	gymnoside I
T_max_	h	0.21 ± 0.08	1.38 ± 0.25	7.50 ± 1.00	1.88 ± 0.25
C_max_	ng/mL	62.31 ± 10.01	620.50 ± 75.98	466.86 ± 96.49	32.82 ± 5.92
AUC_0-t_	h*∗*ng/mL	123.41 ± 9.95	2077.40 ± 358.66	6285.32 ± 1319.90	122.27 ± 9.54
AUC_0-*∞*_	h*∗*ng/mL	140.34 ± 10.02	2116.92 ± 384.37	6316.32 ± 1327.54	123.73 ± 9.81
MRT_0-t_	h	3.29 ± 0.36	2.66 ± 0.50	8.71 ± 0.67	3.28 ± 0.39
MRT_0-*∞*_	h	5.03 ± 0.88	2.88±0.63	8.87 ± 0.68	3.41 ± 0.39

## Data Availability

The data used to support the findings of this study are included within the article.
